# Logical circuits in colloids

**DOI:** 10.1098/rsos.231939

**Published:** 2024-05-22

**Authors:** Nic Roberts, Noushin Raeisi Kheirabadi, Michail-Antisthenis Tsompanas, Alessandro Chiolerio, Marco Crepaldi, Andrew Adamatzky

**Affiliations:** ^1^ Unconventional Computing Laboratory, UWE, Bristol, UK; ^2^ Department of Engineering and Technology, University of Huddersfield, Huddersfield, UK; ^3^ Center for Bioinspired Soft Robotics, Istituto Italiano di Tecnologia, Genova, Italy; ^4^ Electronic Design Laboratory, Istituto Italiano di Tecnologia, Genova, Italy

**Keywords:** unconventional computing, colloids, liquid computers, liquid electronics, liquid robotics

## Abstract

Colloid-based computing devices offer remarkable fault tolerance and adaptability to varying environmental conditions due to their amorphous structure. An intriguing observation is that a colloidal suspension of ZnO nanoparticles in dimethylsulfoxide (DMSO) exhibits reconfiguration when exposed to electrical stimulation and produces spikes of electrical potential in response. This study presents a novel laboratory prototype of a ZnO colloidal computer, showcasing its capability to implement various Boolean functions featuring two, four and eight inputs. During our experiments, we input binary strings into the colloid mixture, where a logical ‘True’ state is represented by an impulse of an electrical potential. In contrast, the absence of the electrical impulse denotes a logical ‘False’ state. The electrical responses of the colloid mixture are recorded, allowing us to extract truth tables from the recordings. Through this methodological approach, we demonstrate the successful implementation of a wide range of logical functions using colloidal mixtures. We provide detailed distributions of the logical functions discovered and offer speculation on the potential impacts of our findings on future and emerging unconventional computing technologies. This research highlights the exciting possibilities of colloid-based computing and paves the way for further advancements.

## 1. Introduction

Unconventional computing involves challenging traditional boundaries in thought, action and computation [1]. Topics within this distinctive field encompass, but are not confined to, the physics of computation, non-classical logics, emerging complexity measures and innovative approaches to hardware, including mechanical, chemical and quantum computing. Practical prototypes and applications arise from the revelation and utilization of principles and mechanisms governing information processing in various systems, including physical, chemical and living systems. This entails the development of efficient algorithms, the design of (almost) optimal architectures and the creation of functional prototypes for future computing devices. Computing with fluids is one of the emerging domains in the field of unconventional computing.

A substance that cannot sustain shear stress when at rest is classified as a fluid: such stress necessarily produces a change in shape and the most remarkable dynamic phenomenon of flow. Specifically, a liquid is categorized as an incompressible fluid. Using liquids as computing devices can be traced back to documented evidence in papers discussing hydraulic algebraic machines [2–4]. In our recent comprehensive overview [5], we thoroughly examine various families of liquid computing devices [6,7]. These include hydraulic mathematical machine integrators, fluid mappers, fluid jets employed in fluidic logic devices to realize logical gates, liquid marble computers and reaction–diffusion computers.

Several years ago, we developed the concept of the liquid cybernetic system [8], a colloidal autonomous system, which is a soft holonomic processor realizing autolographic features [9]. Furthermore, these theoretical ideas of colloid computers were implemented into laboratory prototypes. Our experiments conducted in controlled laboratory conditions also revealed the potential of ZnO colloid mixtures to function as electrical analogue neurons, successfully implementing synaptic-like learning as described in [10,11], as well as demonstrating the manifestation of Pavlovian reflexes [12]. The experimental study presented in [13] showcases the classification capabilities of a Fe_3_O_4_ water-based ferrofluid for digit recognition within an 8 × 8-pixel dataset. Additionally, we demonstrated that this ferrofluid could be programmed using quasi-direct current signals and read in radio frequency mode.

To thoroughly assess the computational capabilities of colloid computers more formally than previously explored, we embarked on a study to determine whether Boolean functions could be straightforwardly implemented in colloid mixtures. To achieve this, we adopted a theoretical approach outlined in [14–16]. The overall idea is explained in table 1, we assume that one stimulating electrode is represented by x and another represented by y . Then, we send binary strings represented by spikes/impulses to colloid and record electrical activity on several output electrodes. In each output electrode, we extract Boolean gates as shown in table 1.

**Table 1.** Representation of gates by combinations of spikes. Black lines show the potential when the network was stimulated by input pair (01), red by Kheirabadi *et al*. [11] and green by Kheirabadi *et al*. [12]. Adopted from Adamatzky *et al*. [15].

spikes	gate	notations
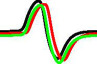	OR	x+y
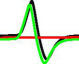	SELECT	y
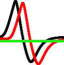	XOR	x⊕y
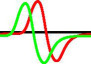	SELECT	x
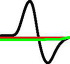	NOT-AND	$\bar{x}y$
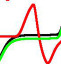	AND-NOT	$x\bar{y}$
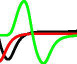	AND	xy

This technique involves selecting a pair of input sites and systematically applying all possible combinations of inputs to these sites, where the electrical characteristics of the input signals represent logical values. The resulting outputs, represented by the electrical responses of the substrate, are recorded on a designated set of output sites [17].

This approach falls within the realm of reservoir computing [18–22] and *in materia* computing [23–27], which are techniques used to analyse the computational properties of physical and biological substrates. Using these methodologies, we aim to gain deeper insights into the computational potential of colloid mixtures in a more rigorous and structured manner.

## 2. Methods

Zinc oxide nanoparticles were purchased from US research nanomaterials. Sodium dodecyl sulphate (SDS) and sodium hydroxide (NaOH) were purchased from Merck. Dimethylsulfoxide (DMSO) pharmaceutical grade 99.9% was purchased from Fisher Scientific. A millipore deionized water generating unit, model Essential, with a resistance of 15 MΩ cm, was used to create deionized water (DIW) in the laboratory . SDS was added to DIW and stirred to get a homogeneous surfactant solution with a concentration of 0.22 wt%. Under stirring, 2 ml of SDS solution and 1 ml of 10 M NaOH were added to the DMSO. The mixture was then treated with 1 mg ZnO nanoparticles while constantly stirring. The resulting dispersion concentration was kept constant at 0.11 mg ml^−1^. For 30 min, the resultant suspension was placed in an ultrasonic bath. The stirring operation was then repeated for a few more hours to achieve a homogeneous dispersion of ZnO [28].

The nanoparticle suspensions were characterized using field emission scanning electron microscopy (FESEM; FEI Quanta 650 FESEM). In this study, the accelerating voltage was set to 10 kV, while the working distance was roughly 5 mm. The contrast and brightness of the photos were adjusted so that particles could be differentiated from the backdrop. An ultraviolet–visible spectrometer (Perkin Elmer Lambda XLS) was used to quantify sample absorbance at room temperature. Dynamic light scattering (DLS) measurements were performed on a Zetasizer Nano ZS (1000 HS, Malvern Instruments Ltd, UK) to analyse the *z*-average hydrodynamic diameter.

**Figure 1.**
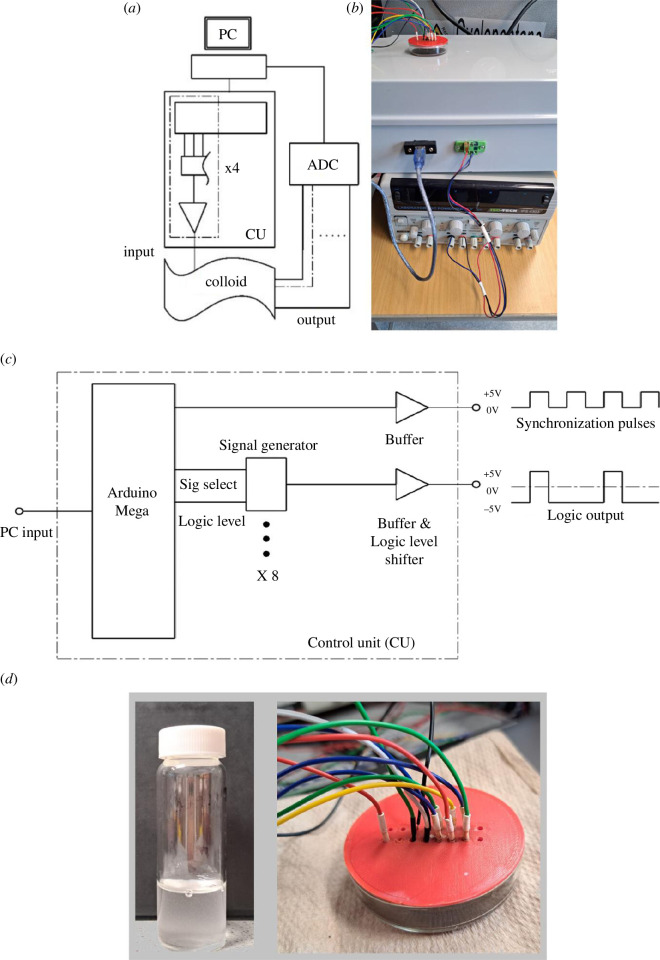
(*a*) A scheme of the experiments. PC—laptop for generating sequences; CU—control unit, the dashed section is a breakdown of a single channel; ADC—analogue to digital converter. Adopted from [29]. (*b*) experimental setup. (*c*) A schematic of the inside of the unit control box. (*d*) A close-up photo of the colloid dish and the electrodes interfacing it.

The developed hardware can send sequences of two-, four- and eight-bit strings to the colloid sample. The strings were encoded as step voltage inputs where −5 V denoted a logical ‘0’ and 5 V a logical ‘1’. The hardware was based around an Arduino Mega 2560 (Elegoo, China) and a series of programmable signal generators, AD9833 (Analog, USA). As depicted in figure 1*a*, a PC is used to program a control unit (CU) and receive the readings from an analogue-to-digital converter (ADC). The CU (depicted in figure 1*b* as the grey box connected to a standard laboratory power supply) is housing the Arduino Mega and multiple AD9833 ICs, as mentioned previously. Because the outputs of Arduino boards are 0–5 V, and the target output is a range of [−5,5] V, the programmable signal generators are used to produce the negative voltage signals. In order to produce the two-, four- and eight-bit strings without redesigning and rewiring the CU, provisions were taken to include multiple programmable signal generators (as abstracted in figure 1*c*, where only one of them and its output are depicted for simplicity reasons). The activation of these signal generators is controlled through the Arduino Mega (also depicted in figure 1*c* within the CU entity) that is programmed through the PC.

To search for two-, four- and eight-input Boolean circuits, we used two-, four- and eight-input electrodes, respectively. The input electrodes were 10 µm diameter platinum rods inserted into the colloid container with a separation of 5 mm between them. Data acquisition (DAQ) probes were placed in a parallel line, separated by 5 mm. There were two DAQ differential outputs from the sample container inputted to a Pico 24 (Pico Technology, UK) ADC. The third channel was used to pass a pulse to the ADC on every input state change. See figure 1 for a schematic of the apparatus. Note here that the inputs to the colloid sample can be two, four or eight electrodes (only one illustrated in figure 1*a* for simplicity reasons), whereas the output of the colloid is a one-bit signal that is measured by a differential pair of electrodes (as sketched in figure 1*a*). There were a total of 138 repeats.

A sequence of two-, four- or eight-bit strings counting up from binary *00* to *11*, *0000* to *1111* or *00000000* to *11111111* with a state change every 15 s, were passed into the colloid. Namely, all possible states for the available electrodes were tested. For the two-bit case, the state of the inputs is sequentially altered every 15 s between *00*, *01*, *10* and *11*. Similarly, all possible states of the four- and eight-bit strings are sequentially applied to the sample. Samples from two channels were taken at 1 Hz over the whole duration of a given experimental run. Peaks for each channel were located for a set of 10 thresholds, from 100 to 600 mV with step 50 mV, for each input state, *0000* to *1111*.

## 3. Results

### 3.1. Colloid structural characteristics

**Figure 2.**
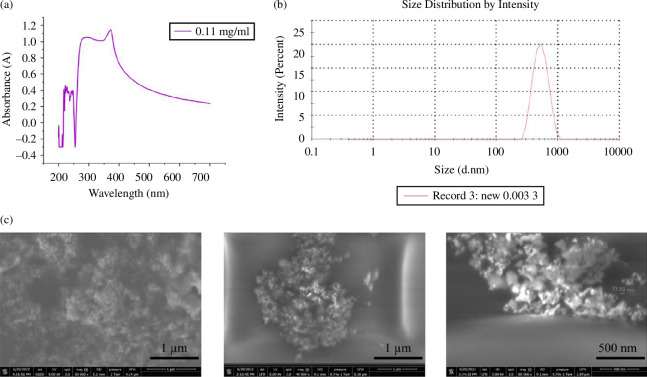
Structural characterization of colloid used in experiments. (*a*) UV–visible spectra of the ZnO colloid. Adopted from [10]. (*b*) Particle size distribution of ZnO nanoparticles in colloidal solution using DLS. (*c*) SEM images of drop-cast ZnO colloids on a copper substrate in different magnifications. Adopted from [12].

The absorption spectrum of a ZnO colloid, with a concentration of 0.11 mg ml^−1^, was measured at room temperature using UV–visible spectroscopy. The recorded spectrum covers a wavelength range of 200–700 nm. Figure 2*a* illustrates the UV–visible absorption spectrum plot. The spectrum shows a prominent peak at 372 nm, indicating hexagonal ZnO nanoparticles [30]. Comparing these findings with existing literature, there is a strong agreement with previous reports [31,32]. The optical band gap was calculated using the following equation:


$$E_{g}(eV)=hc/\lambda =1240/\lambda .$$

In this equation, $E_{g}$ represents the optical band gap, *h* is Planck’s constant, *c* is the speed of light and λ is the wavelength corresponding to the maximum absorption. The calculated value for the optical band gap is 3.35 eV, which aligns closely with previous findings from other sources [31,33,34].

DLS was used to characterize the ZnO nanoparticles in the colloid. Figure 2*b* displays the size distribution of these nanoparticles. A particle’s average gyration (hydrodynamic) diameter is determined to be 496 nm, nearly 20 times the average diameter of an individual particle. As an amphoteric oxide, ZnO undergoes hydrolysis when exposed to water, forming a hydroxide coating on its surface. This coating contributes to an increase in the hydrodynamic diameter of the particles [35].

A thin layer of ZnO colloid was prepared to analyse the particles’ morphology and size using the FESEM technique. This was done by drop-casting a drop of ZnO particle suspension, with a concentration of 0.11 mg ml^−1^, onto a copper foil with a 100 µm thickness. The preparation was carried out at room temperature.

The FESEM results, as shown in figure 2*c*, reveal the occurrence of particle agglomeration during the sample preparation process. Due to the surface tension of the solvent as it evaporates, the FESEM observations rarely display individual, separated spheres. Instead, most of the ZnO spheres appear to be multi-layered. This can be attributed to the increased liquid surface tension, which draws the nanoparticles closer and leads to their re-aggregation during the drying process [36].

### 3.2. Extracting Boolean gates

Boolean strings were extracted from the data, where a logic ‘1’ was noted for a channel if it had a peak outside the threshold band for a particular state. Otherwise, a value of ‘0’ was recorded, and the peak’s polarity was not considered.

The strings for each experimental repeat were stored in their respective Boolean table. To extract state graphs, a state/node was defined as the string of output values from each channel at each input state, and transitions/edges were defined as a change in the input state. This led to a total (500 + 470 + 410 = 1380) state graphs. The sum of products (SOP) Boolean functions were calculated for the output channel. For each repetition, we collected data and applied 10 thresholds, giving 1380 individual truth tables.

**Figure 3.**
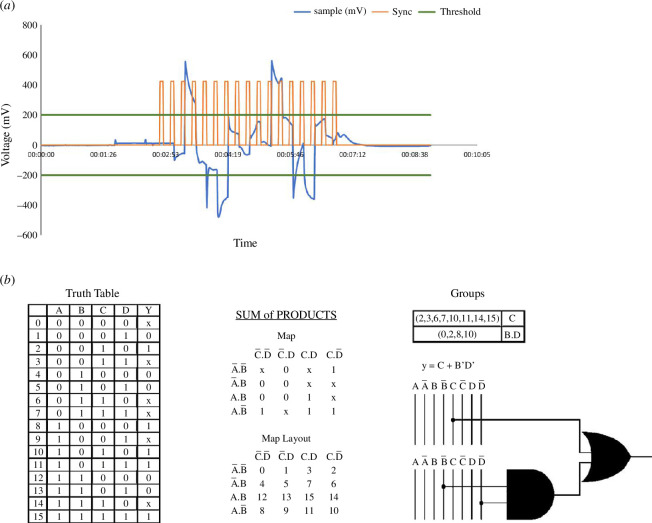
Workflow example. (*a*) The voltage measurements of the sample. The DAQ are in blue. The synchronization signal is in orange, the threshold band is green. (*b*) The resulting truth table and the extracted function.

SOP extraction is depicted in figure 3. If a peak is discovered during an input state, it is considered a logical ‘1’. The DAQ measurements are shown by a blue line. The synchronization signal is illustrated as an orange line, indicating the state change. The threshold band is the green line, while peaks outside of it are marked with ‘x’ (figure 3, b). The resulting truth table is then reduced to the sum of products as depicted in figure 3.

**Figure 4.**
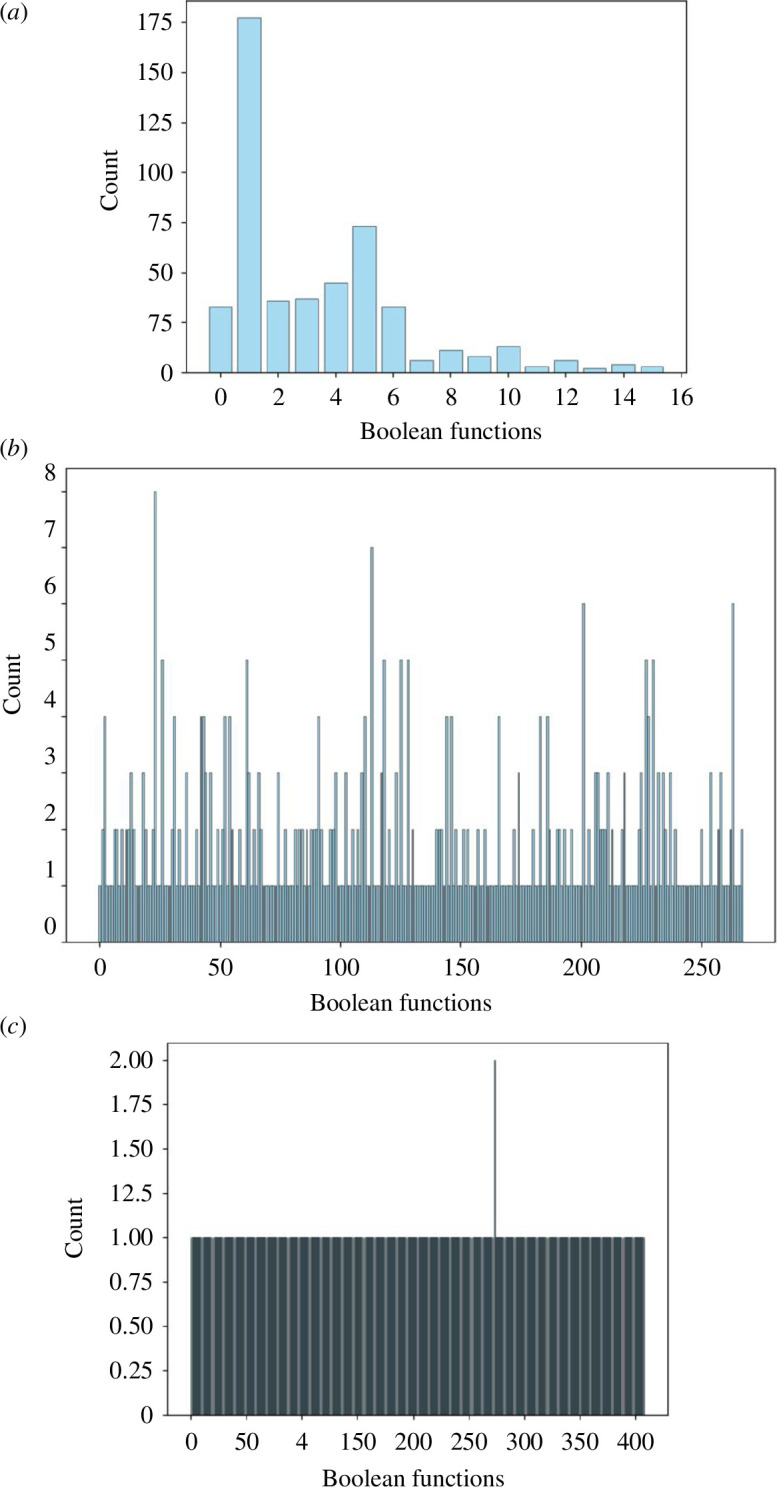
Distribution of occurrences of two-input (*a*), four-input (*b*) and eight-input (*c*) Boolean gates discovered in laboratory experiments with colloids. The horizontal axis depicts Boolean functions in decimal codification. The vertical axis is the number of detected gates.

**Figure 5.**
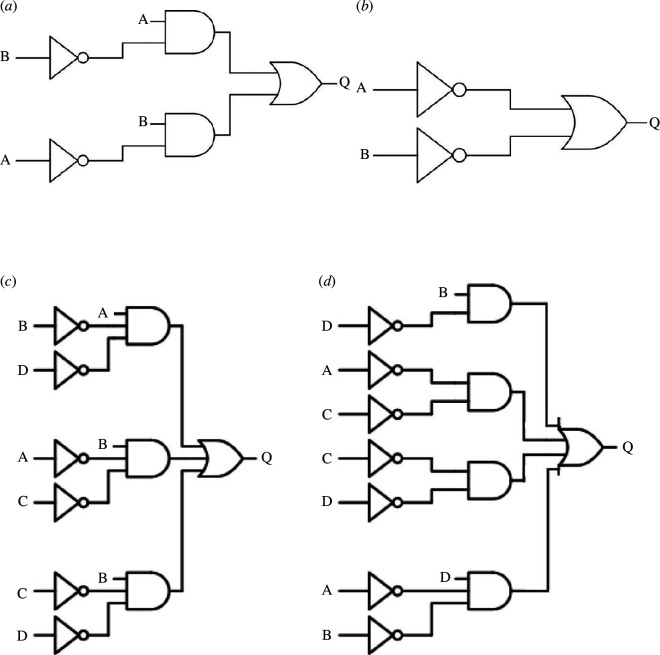
Examples of logical circuits realized in (*a*,*b*) two-input and (*c*,*d*) four-input logical circuits.

We have discovered a wide range of Boolean gates. Distributions of gates are shown in figure 4. Those frequently found in experiments with two-input gates are shown in table 2 and illustrated in terms of circuits in figure 5*a*,*b*. The most common gate is $\bar{A}+\bar{B}$ , which is a NAND gate, a logic gate producing an output that is False only if all its inputs are True; thus, its output is a complement to that of an AND gate. The NAND gate is followed by OR gate and then by two NOT-AND gates.

**Table 2.** The most commonly found two-input Boolean functions, n is a frequency of the functions’ discovery.

gate	n
$\bar{A}+\bar{B}$	73
A+B	45
$\bar{A}+B$	37
$A+\bar{B}$	33
A⋅B	8
$B\cdot \bar{A}$	6
$\left(A\cdot \bar{B})+(B\cdot \bar{A}\right)$	4
$\left(A\cdot B)+(\bar{A}\cdot \bar{B}\right)$	3
$A\cdot \bar{B}$	3
$\bar{A}\cdot \bar{B}$	2

The most frequent four-input gates are shown in table 3 and illustrated with example circuits in figure 5*c*,*d*. The size of a Boolean circuit is defined as the number of gates in the circuit. Among the most frequent four-input circuits (table 3), the smallest circuits are $A\cdot \bar{B}\cdot \bar{C}\cdot D$ and $A\cdot \bar{B}\cdot \bar{C}\cdot \bar{D}$ , and the largest one is $\left(A\cdot D\cdot \bar{B})+(B\cdot D\cdot \bar{A})+(A\cdot \bar{B}\cdot \bar{C})+(B\cdot \bar{A}\cdot \bar{C})+(D\cdot \bar{A}\cdot \bar{C}\right)$ .

**Table 3.** The most commonly found four-input Boolean functions, n is a frequency of the functions’ discovery.

gate	n
$\left(A\cdot \bar{B})+(B\cdot \bar{A}\cdot \bar{C})+(B\cdot \bar{C}\cdot \bar{D}\right)$	7
$\left(C\cdot D\cdot \bar{B})+(A\cdot \bar{B}\cdot \bar{D})+(B\cdot \bar{A}\cdot \bar{D})+(D\cdot \bar{A}\cdot \bar{C}\right)$	6
$\left(A\cdot \bar{B}\cdot \bar{D})+(B\cdot \bar{A}\cdot \bar{C}\cdot \bar{D}\right)$	6
$\left(\bar{A}\cdot \bar{D})+(A\cdot B\cdot C\cdot D)+(B\cdot \bar{A}\cdot \bar{C})+(C\cdot \bar{A}\cdot \bar{B}\right)$	5
$\left(A\cdot \bar{B}\cdot \bar{D})+(B\cdot \bar{A}\cdot \bar{C})+(B\cdot \bar{C}\cdot \bar{D}\right)$	5
$A\cdot D\cdot \bar{B}\cdot \bar{C}$	5
$A\cdot \bar{B}\cdot \bar{C}\cdot \bar{D}$	5
$\left(B\cdot C\cdot D)+(B\cdot C\cdot \bar{A})+(C\cdot D\cdot \bar{A})+(A\cdot \bar{B}\cdot \bar{C}\cdot \bar{D}\right)$	5
$\left(A\cdot D\cdot \bar{B})+(B\cdot D\cdot \bar{A})+(A\cdot \bar{B}\cdot \bar{C})+(B\cdot \bar{A}\cdot \bar{C})+(D\cdot \bar{A}\cdot \bar{C}\right)$	5
$\left(D\cdot \bar{A})+(D\cdot \bar{B})+(B\cdot \bar{A}\cdot \bar{C}\right)$	5

With regard to eight-input gates, all discovered gates are unique, i.e. have been measured just once. Only the following function has been found twice:


$$\left(A\cdot B\cdot F\cdot \bar{C}\cdot \bar{E})+(A\cdot D\cdot F\cdot \bar{C}\cdot \bar{E})+(A\cdot G\cdot H\cdot \bar{B}\cdot \bar{C})+(B\cdot D\cdot E\cdot \bar{A}\cdot \bar{F})+(B\cdot E\cdot H\cdot \bar{A}\cdot \bar{C})+(C\cdot D\cdot E\cdot \bar{B}\cdot \bar{F})+(D\cdot E\cdot H\cdot \bar{B}\cdot \bar{G})+(D\cdot F\cdot H\cdot \bar{A}\cdot \bar{B})+(B\cdot C\cdot D\cdot F\cdot G\cdot \bar{H})+(B\cdot C\cdot D\cdot G\cdot H\cdot \bar{F})+(B\cdot E\cdot \bar{A}\cdot \bar{G}\cdot \bar{H})+(C\cdot F\cdot \bar{B}\cdot \bar{G}\cdot \bar{H})+(E\cdot H\cdot \bar{A}\cdot \bar{C}\cdot \bar{F})+(F\cdot H\cdot \bar{B}\cdot \bar{D}\cdot \bar{E})+(A\cdot C\cdot E\cdot G\cdot \bar{B}\cdot \bar{D})+(A\cdot E\cdot F\cdot G\cdot \bar{C}\cdot \bar{D})+(B\cdot D\cdot E\cdot G\cdot \bar{C}\cdot \bar{F})+(B\cdot E\cdot F\cdot G\cdot \bar{A}\cdot \bar{D})+(C\cdot F\cdot G\cdot H\cdot \bar{D}\cdot \bar{E})+(A\cdot C\cdot D\cdot E\cdot F\cdot H\cdot \bar{G})+(B\cdot \bar{C}\cdot \bar{E}\cdot \bar{G}\cdot \bar{H})+(C\cdot \bar{B}\cdot \bar{D}\cdot \bar{F}\cdot \bar{G})+(D\cdot \bar{C}\cdot \bar{E}\cdot \bar{F}\cdot \bar{H})+(E\cdot \bar{A}\cdot \bar{B}\cdot \bar{D}\cdot \bar{H})+(E\cdot \bar{B}\cdot \bar{D}\cdot \bar{G}\cdot \bar{H})+(B\cdot C\cdot D\cdot \bar{A}\cdot \bar{E}\cdot \bar{G})+(B\cdot D\cdot F\cdot \bar{C}\cdot \bar{G}\cdot \bar{H})+(B\cdot D\cdot G\cdot \bar{A}\cdot \bar{C}\cdot \bar{E})+(B\cdot E\cdot H\cdot \bar{C}\cdot \bar{F}\cdot \bar{G})+(C\cdot D\cdot G\cdot \bar{B}\cdot \bar{E}\cdot \bar{H})+(C\cdot E\cdot F\cdot \bar{D}\cdot \bar{G}\cdot \bar{H})+(C\cdot G\cdot H\cdot \bar{A}\cdot \bar{E}\cdot \bar{F})+(D\cdot E\cdot F\cdot \bar{A}\cdot \bar{B}\cdot \bar{C})+(B\cdot D\cdot \bar{E}\cdot \bar{F}\cdot \bar{G}\cdot \bar{H})+(B\cdot F\cdot \bar{A}\cdot \bar{D}\cdot \bar{E}\cdot \bar{G})+(C\cdot D\cdot \bar{A}\cdot \bar{B}\cdot \bar{E}\cdot \bar{H})+(C\cdot H\cdot \bar{D}\cdot \bar{E}\cdot \bar{F}\cdot \bar{G})+(A\cdot C\cdot E\cdot G\cdot \bar{D}\cdot \bar{F}\cdot \bar{H})+(A\cdot \bar{B}\cdot \bar{C}\cdot \bar{F}\cdot \bar{G}\cdot \bar{H})+(D\cdot \bar{A}\cdot \bar{B}\cdot \bar{C}\cdot \bar{E}\cdot \bar{G})+(G\cdot \bar{A}\cdot \bar{C}\cdot \bar{D}\cdot \bar{E}\cdot \bar{H}\right)$$

## 4. Discussion

Our laboratory experiments successfully demonstrated the feasibility of implementing a wide range of many-input logical gates within a colloid mixture comprising ZnO nanoparticles. The discovered two-input gates exhibit functional completeness, enabling the implementation of arbitrary Boolean functions. Notably, the four- and eight-input functions discovered showcase a remarkable level of nonlinearity, suggesting that the dynamical behaviour of colloid-based logical devices could be characterized by multiple attractors and bifurcation points, more than features such as resistive switching, which was already observed [37].

While the True mechanisms of Boolean frequency profiles still remains unknown, we can speculate that the observed distribution of frequencies in colloidal logical gates based on electrical impulses may be attributed to certain physicochemical features of the colloidal system. The first possible reason is particle interaction and aggregation. In fact, colloids consist of suspended particles in a medium. The specific interactions between these particles can influence the formation of logical gates. For instance, the higher frequency of $\bar{A}+\bar{B}$ and A+B gates could result from favourable particle interactions that lead to stable aggregates under the given electrical impulses. The second reason could be in surface chemistry. Certain chemical functional groups on the particle surfaces may promote or inhibit the formation of specific logical gates. The preference for certain gates might be linked to the ease with which particles with complementary surface chemistries come together under the applied electrical conditions. One such example of an interfacial coupling mechanism can be found in previous work using ZnO nanoparticles embedded in an oligomeric/polymeric matrix where ethoxylated groups could chemically react with the oxygen vacancies found at the surface of the nanoparticles [37]. This reversible mechanism allowed the stable realization of a logic state, read as a current or resistance, programmable through the use of voltage pulses of suitable sign, amplitude and duration. The third reason might be attributed to electric field effects. The distribution of electrical charges on colloidal particles can affect their movement, statistical properties and interaction. The specific configuration of electrical impulses may favour the alignment or aggregation of particles in ways that correspond to the more frequent logical gates. Variations in electric field strength or polarity could influence the likelihood of certain gate formations.

The results reveal the remarkable fault tolerance and adaptability of colloid-based computing devices, owing to their amorphous structure. The ZnO colloidal suspension exhibited reconfiguration and produced spikes of electrical potential in response to electrical stimulation. This discovery highlights the exciting possibilities of colloid-based computing and its potential impact on future and emerging unconventional computing technologies.

One of the key aims of future studies would be the reprogramming of colloid computers. To reprogram colloid computers, one can alter the configuration of particles within the colloid. Several methods for achieving this reconfiguration include manipulating electric/magnetic fields both steady and oscillating [38], adjusting particle size, changing surface chemistry, applying magnetic forces or varying the temperature of the colloid. Each of these approaches offers unique means to control and modify the arrangement of particles, thereby influencing the computational behaviour of the colloid system.

Another future target could be scaling up the colloid computers, which can be implemented by several techniques and combinations of them. The first approach would be to expand the number of colloidal particles within the system. This may involve optimizing production processes to generate larger quantities of colloids, ensuring stability and uniformity in their properties. The second technique could improve precision in manipulating individual colloidal particles. Advanced techniques in particle synthesis, manipulation and assembly can contribute to more reliable and scalable systems. The third approach is seen in parallelization—implementation of parallel processing by incorporating multiple colloid computing units. This approach involves running several computations simultaneously, enhancing computational throughput and efficiency. The fourth approach would be in the design of multi-level logical circuits by cascading colloid droplets. This allows for more complex computations and increased computational capacity within a single system. The fifth technique could be in modulating input signals to convey equivalent inline and just-in-time executed programs. This concept aligns with achieving functionalities similar to microprogrammed solid-state computers. The sixth approach encompasses the introduction of new physics. Colloidal suspensions are intrinsically capable of showing long-range features involving classical and quantum interactions. The introduction of new physics in information processing could open unforeseen routes to high-performance computing. By combining these approaches, we can work towards scaling up colloid computers, unlocking their potential for larger and more powerful computational tasks. Looking ahead, our future research direction could focus on cascading the colloid droplets to construct multi-level logical circuits. Additionally, we aim to develop protocols for programming dynamical logical circuits within the colloid droplets. Leveraging on the observed multi-stability that is the presence of multiple attractors, a possible blue sky objective would regard the implementation of a sequential computing machine, which, similarly to solid-state computers, can be programmed and can execute instructions. Our laboratory experiments have already demonstrated the fundamental assumptions needed to reach this goal, showcasing the possibility of implementing in-memory computing with ferrofluids, therefore showing the coexistence of memorizing and computing capabilities. For the particular case studied here, the presence of multiple Boolean transfer functions inherently suggests the existence of a pre-built program in the colloid, here, a function of threshold, but in general, a function of other parameters, including time. A possible solution to achieve functionalities similar to a microprogrammed solid-state computer could regard modulating input signals to convey an equivalent inline and just-in-time executed program. Further studies can then focus on the stimulations’ meaning and timing and the feasible techniques for their modulation. This concept, *inter alia*, further overlaps with the field of neuromorphic computing because inputs can degenerate into spikes for low-duty cycles. These advancements would enhance the capabilities and expand the potential applications of colloid-based computing systems.

## 5. Conclusion

In conclusion, this study presents a novel laboratory prototype of a ZnO colloidal computer and demonstrates its capability to implement various Boolean functions with two, four and eight inputs. Through experiments using binary strings as input and recording the electrical responses of the colloid mixture, we successfully extracted truth tables and implemented a wide range of logical functions.

The extraction of Boolean gates from the recorded data resulted in the discovery of a wide range of gates, including frequently found two-input and four-input gates. The distribution of gates showcased the nonlinearity and complexity of the colloid-based logical devices. These findings suggest the potential existence of multiple attractors and bifurcation points within the colloid mixture, indicating its dynamic behaviour and computational capabilities.

Overall, this study contributes to the understanding and advancement of colloid-based computing systems, highlighting their potential for unconventional computing paradigms and paving the way for further advancements in the field.

## Data Availability

The datasets made and analysed during the current study are available in the Zenodo repository under [39].
